# Diverse mycorrhizal maize inbred lines differentially modulate mycelial traits and the expression of plant and fungal phosphate transporters

**DOI:** 10.1038/s41598-022-25834-7

**Published:** 2022-12-08

**Authors:** Luca Giovannini, Cristiana Sbrana, Manuela Giovannetti, Luciano Avio, Alessandra Lanubile, Adriano Marocco, Alessandra Turrini

**Affiliations:** 1 Department of Agriculture, Food and Environment, University of Pisa, Via del Borghetto 80, 56124 Pisa, Italy; 2 Institute of Agricultural Biology and Biotechnology, National Research Council of Italy, Via Moruzzi 1, 56124 Pisa, Italy; 3 Department of Sustainable Crop Production, Università Cattolica del Sacro Cuore, Via Emilia Parmense 84, 29122 Piacenza, Italy

**Keywords:** Microbiology, Plant sciences

## Abstract

Food production is heavily dependent on soil phosphorus (P), a non-renewable mineral resource essential for plant growth and development. Alas, about 80% is unavailable for plant uptake. Arbuscular mycorrhizal fungi may promote soil P efficient use, although the mechanistic aspects are yet to be completely understood. In this study, plant and fungal variables involved in P acquisition were investigated in maize inbred lines, differing for mycorrhizal responsiveness and low-P tolerance, when inoculated with the symbiont *Rhizoglomus irregulare* (synonym *Rhizophagus irregularis*). The expression patterns of phosphate transporter (*PT*) genes in extraradical and intraradical mycelium (ERM/IRM) and in mycorrhizal and control maize roots were assessed, together with plant growth responses and ERM extent and structure. The diverse maize lines differed in plant and fungal accumulation patterns of *PT* transcripts, ERM phenotypic traits and plant performance. Mycorrhizal plants of the low-P tolerant maize line Mo17 displayed increased expression of roots and ERM *PT* genes, compared with the low-P susceptible line B73, which revealed larger ERM hyphal densities and interconnectedness. ERM structural traits showed significant correlations with plant/fungal expression levels of *PT* genes and mycorrhizal host benefit, suggesting that both structural and functional traits are differentially involved in the regulation of P foraging capacity in mycorrhizal networks.

## Introduction

World population is expected to reach 9.7 billion by 2050 and 10.9 billion in 2100^1^ with a consequent increase in food demand, whose sustainable production will represent a pivotal challenge for agriculture. Current food crop production systems strongly depend on the use of chemical fertilizers, among which phosphorus (P), essential for plant growth and development, represents a non-renewable resource, whose reserves in mined rocks are estimated to decline within 100–200 years^2^.

P is a key macronutrient for plants, being involved in many biological processes, such as respiration, photosynthesis, phospholipid biosynthesis and in phosphorylation-based signaling mechanisms^3^. As a central component of nucleic acids, membrane phospholipids and energy metabolism in the form of ATP, P represents up to 0.2% of plant dry weight^4^. Plants absorb P as inorganic phosphate (Pi), but, although the total amount of P in the soil may be high, more than 80% is unavailable for plant uptake, being present in organic pool or immobilized by cations and clays or lost, eventually causing water eutrophication^5^. Accordingly, the development of strategies for enhancing P availability from the soil, reducing external P input in agricultural ecosystems, is essential for the sustainable production of food crops. Among plant root-associated fungal microbiota, arbuscular mycorrhizal (AM) fungi (AMF) can be exploited for this purpose^6,7^.

AMF are an ancient group of soil fungi belonging to the phylum Glomeromycota^8^, which establish beneficial associations with the roots of about 80% of plant species, including the most important food crops. AMF are key organisms in agricultural ecosystems, increasing plant performance and fitness and enhancing tolerance to different stress conditions in the most diversified environments^9,10^. AMF deliver also important ecosystem services, such as soil structure and carbon sequestration in the soil^11,12^, contributing to global warming mitigation^13^. In this mutualistic symbiosis AMF obtain carbon compounds by plants, in exchange of mineral nutrients—mainly P, N, S, K, Ca, Cu Zn—which are absorbed and translocated through a highly interconnected extraradical mycelium (ERM) that spreads in the soil from colonized roots^14^. ERM, extending up to 25 m g^−1^ soil^15^, improves the absorption of those soil nutrients which are out of reach or not accessible to the host plants. Such mycorrhizal P absorption pathway is often more effective than the direct uptake pathway through epidermal root cells^16^. So far, only a few phosphate transporter (*PTs*) genes have been characterized in AMF: one in *Glomus versiforme* (*GvPT*^17^), *Funneliformis mosseae* (formerly *Glomus mosseae*, *GmosPT*^18^) and *Gigaspora margarita* (*GigmPT*^19^) and seven in *Rhizoglomus irregulare* (formerly *Glomus intraradices*, synonym *Rhizophagus irregularis RiPT1*-*RiPT7*^20,21^). Different studies reported that such transporters are expressed in ERM and in arbusculated cells^18–20,22,23^, demonstrating both AMF ability to acquire P from soil and to control P fluxes by intraradical mycelium (IRM). The final translocation to the host is carried out by specific plant PTs of the Phosphate transporter 1 (Pht1) family, identified in different plant species, including barrel medic, potato, tomato, rice and maize^24^. The transcription of *Pht1* genes in the different species is modulated by external P availability and was found to be regulated or induced in mycorrhizal roots^25–31^.

Among the most important plant species cultivated worldwide, maize includes many varieties selected under sufficient input of P fertilization, potentially limiting plant susceptibility to mycorrhizal colonization. Several studies stressed the importance of screening a high number of genotypes, in order to assess the genetic variability of plant susceptibility to mycorrhizal colonization^32–37^. In maize only two studies reported a wide characterization of inbred lines able to grow in low-P soil conditions and to respond to mycorrhizal inoculation^32,38^. Studies aimed at evaluating the expression of fungal and plant P transporters in different lines of the same plant species represent a major challenge for the selection of plant and fungal genotypes to be used in sustainable crop production systems. Notwithstanding, scanty information is available on the role played by host plant genotype in the regulation of fungal P uptake and ERM growth and architecture. The expression of different fungal and plant *PTs* in ERM and mycorrhizal roots has been evaluated in a few species (*Populus trichocarpa*, *Sorghum bicolor*, *Medicago truncatula* and *Zea mays*^23,38,39^), while the differential expression patterns of plant *PTs* in different genotypes of the same species were assessed only in maize and wheat plants^38,40^.

In this work, with the aim of studying how plant genotypes modulate plant and fungal variables involved in P acquisition at low external P availability, four maize inbred lines, previously reported to differ for mycorrhizal responsiveness and low-P tolerance^32,38^, were grown in symbiosis with the AM fungal species *Rhizoglomus irregulare* (basionym *Glomus irregulare*, synonym *Rhizophagus irregularis*) in an in vivo experimental system. Such microcosm experimental system was specifically devised for the assessment of ERM molecular and structural traits, meanwhile allowing the detection of mycorrhizal host benefits and plant gene expression. To this aim, the maize lines B73 and Oh43 (characterized by high mycorrhizal responsiveness and low tolerance to P deficiency), Oh40B and Mo17 (characterized by low mycorrhizal responsiveness and high tolerance to P deficiency)^32,38^ were evaluated for (i) the expression patterns of fungal and plant *PTs* in ERM, IRM and mycorrhizal roots, and (ii) phenotypic traits related to host growth and P acquisition, and to ERM extent and structure.

## Results

### Expression of *Z. mays Pht1* (*ZmPht1*) genes in different maize inbred lines

**Figure 1:**
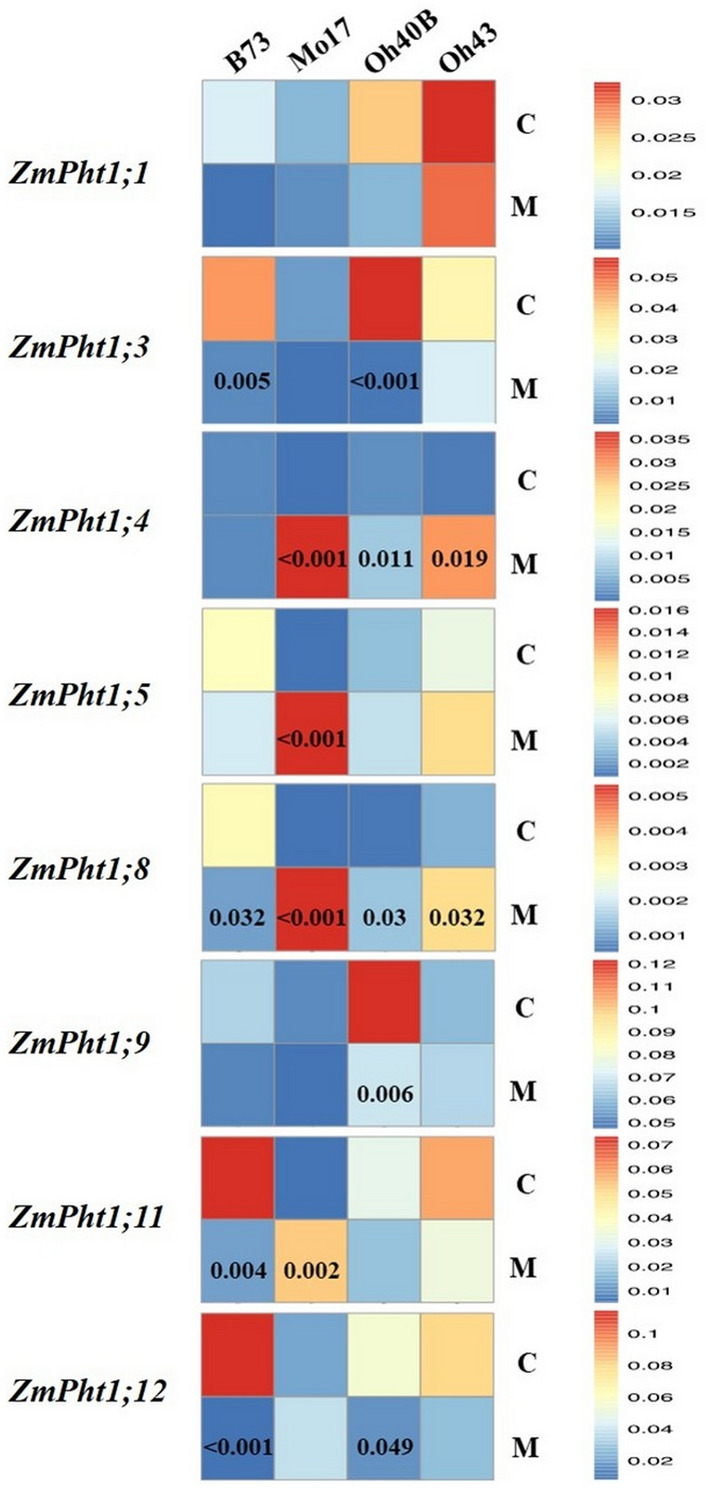
Heat map based on normalized expression of P transporter (*ZmPht1*) transcripts in roots of *Zea mays* plants belonging to four inbred lines, in symbiosis (M) or not (C) with *Rhizoglomus irregulare*, under low phosphorus availability (0.47 mg kg^−1^). Mean gene expression was scaled independently for each phosphate transporter from blue (minimum) to red (maximum). Significant differences between treatments for each phosphate transporter are indicated by the relevant *P* values (Tukey’s HSD post hoc test). Expression levels were normalized against the *ZmActin1* gene.

**Figure 2:**
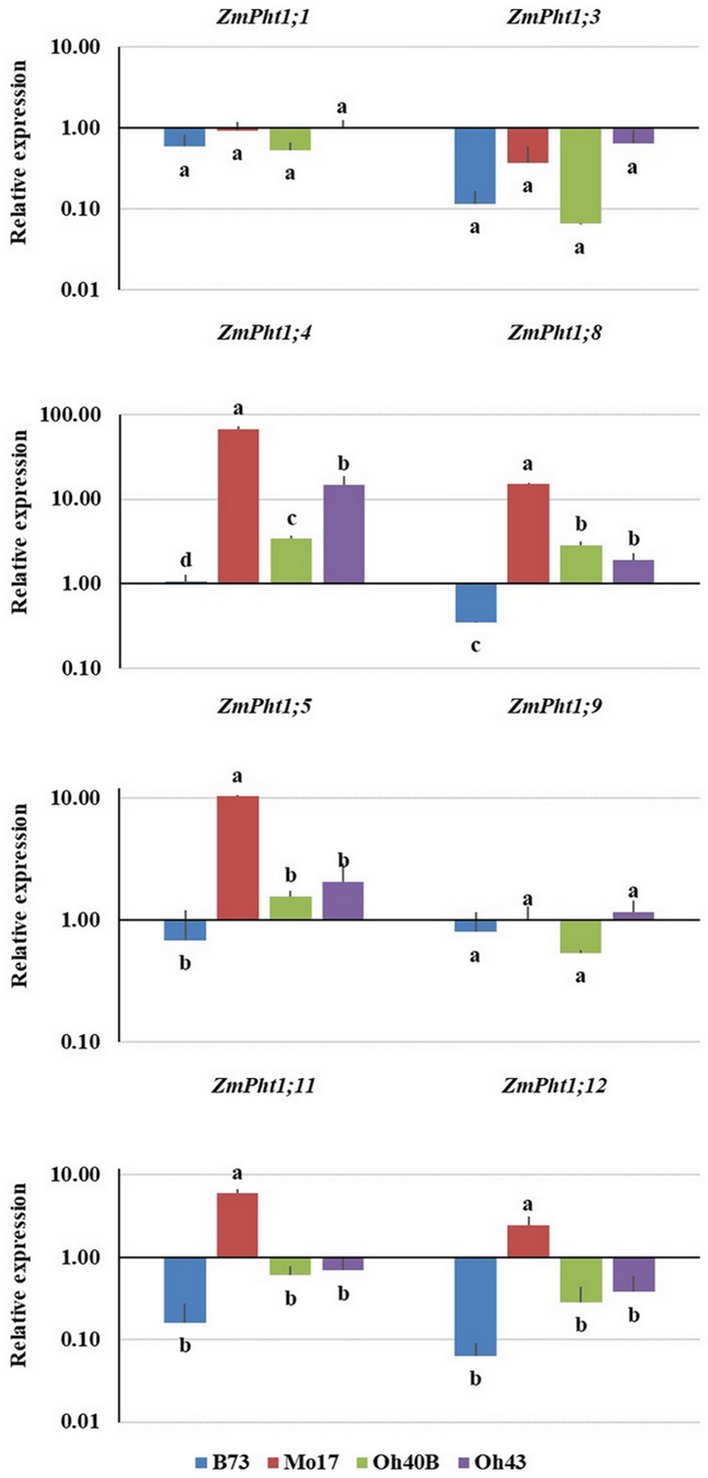
Normalized relative expression of phosphate transporters (*ZmPht1*) in mycorrhizal roots of *Zea mays* plants belonging to four inbred lines, grown in symbiosis with *Rhizoglomus irregulare*, under low phosphorus availability (0.47 mg kg^−1^). Data are presented as mean values ± SEM (n = 6). For each transporter gene, same letters indicate no significant differences (*P* > 0.05) between maize lines by Tukey’s HSD or Tamhane T2 test. All expression levels were normalized against the *ZmActin1* gene.

Using RT-qPCR the relative expressions of eight out of 13 *ZmPht1* genes were reliably quantified in control and mycorrhizal maize roots (Figs. 1 and 2). *ZmPht1*s transcription levels of control plants varied among lines, showing the lowest values in Mo17 (see Supplementary Tables S1 and S2 online). Compared with Mo17, control plants of the other lines showed significantly higher expression levels of transcripts of *ZmPht1;3* (in all lines), of *ZmPht1;8*, *ZmPht1;11* and *ZmPht1;12* (in B73) and of *ZmPht1;9* (in Oh40B) (see Supplementary Table S1 online).

**Figure 3:**
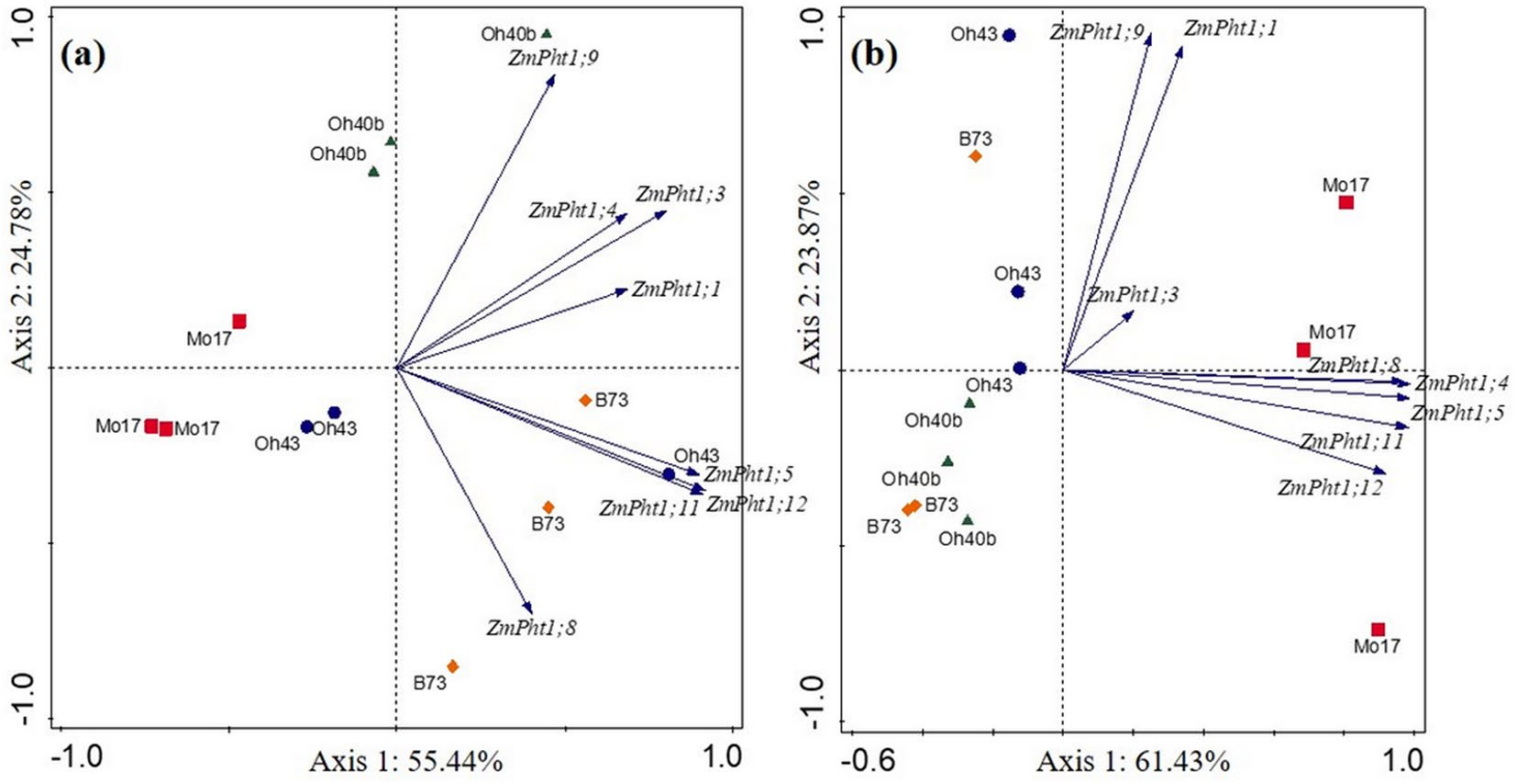
Principal Component Analysis (PCA) biplots of the normalized expression level of P transporters (*ZmPht1*) expressed in roots at low phosphorus availability (0.47 mg kg^−1^) by four inbred lines of *Zea mays* either uninoculated (**a**) or in symbiosis with *Rhizoglomus irregulare* (**b**). In (**a**), the first and second axes explain 80.22% of total variance; in (**b**), the first and second axes explain 85.30% of total variance.

In mycorrhizal plants transcription levels were also different among lines. The analysis of relative expression data of mycorrhizal maize B73 indicated significantly lower expression for *ZmPht1;3*, *ZmPht1;8*, *ZmPht1;11* and *ZmPht1;12* compared with controls (Fig. 1). Moreover, in mycorrhizal B73 line, root expression was lower compared with most of the other lines, and significantly lower for the transporter genes *ZmPht1;4* and *ZmPht1;8* (*P* < 0.05) (Fig. 2). On the contrary, the inbred line Mo17 showed an opposite trend, significantly upregulating half of the assayed P transporter genes under AMF colonization, compared with controls (*ZmPht1;4*, *ZmPht1;5*, *ZmPht1;8* and *ZmPht1;11*, Fig. 1). Interestingly, mycorrhizal Mo17 plants showed significantly higher expression in 5 out of 8 transporters, compared with those of B73 (Fig. 2). Mycorrhizal plants belonging to lines Oh40B and Oh43 showed variable expression levels, depending on the transporters (Figs. 1 and 2). Normalized datasets of *ZmPht1*s expression in control and mycorrhizal plants were separately submitted to PCA to highlight the differences in transcription patterns among the four lines: in the absence of mycorrhizal symbiosis, expression data of *ZmPht1;5*, *ZmPht1;8*, *ZmPht1;11* and *ZmPht1;12* were highly related to maize line B73 (Fig. 3a). In mycorrhizal plants, an opposite relationship between *ZmPht1*s expression and maize lines was shown, as most transporters were more related to Mo17, compared with the other lines (Fig. 3b).

### Expression of *R. irregulare* PT (*RiPT*) genes in IRM and ERM in mycorrhizal maize inbred lines

**Figure 4:**
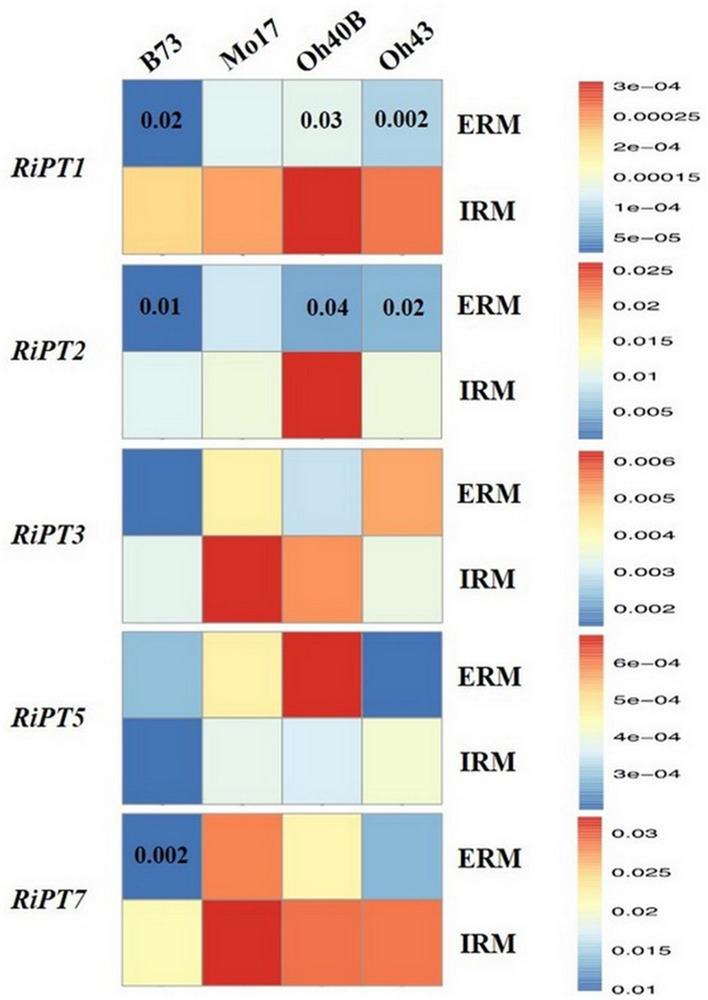
Heat map based on qRT-PCR quantification of expression levels of *Rhizoglomus irregulare* phosphate transporters (*RiPT*) in extraradical (ERM) and intraradical (IRM) mycelium under low phosphorus availability (0.47 mg kg^−1^). Mean gene expression was scaled independently for each phosphate transporter from blue (minimum) to red (maximum). For each *RiPT* and maize line, significant differences between values obtained from the two mycelial types are indicated by the relevant *P* value. Expression levels were normalized against the *ef1-α* gene.

The relative expression of five *RiPT*s (*RiPT1*, *RiPT2*, *RiPT3*, *RiPT5* and *RiPT7*) was analyzed in ERM and IRM of mycorrhizal maize plants belonging to the different inbred lines (Fig. 4, Supplementary Table S3 online). *RiPT*s expression in ERM growing from B73 colonized roots mostly showed lower values compared with those of ERM associated to the other maize lines. On the contrary, the ERM growing from Mo17 roots showed the highest relative expression levels detected among maize lines. As to IRM, the expression of the five *RiPT*s did not show significant differences among the four maize lines (see Supplementary Table S3 online).

The expression levels of individual *RiPT*s, mostly lower in ERM compared with IRM, were not correlated in the two fungal structures, as indicated by a not significant Mantel’s correlation test (*P* = 0.62), obtained when comparing the two sets of expression data. Actually, a differential expression of the five *RiPT*s between ERM and IRM was detected within each inbred line, with significantly lower expression of *RiPT1* and *RiPT2* in ERM produced in symbiosis with three out of four maize lines (Fig. 4, Supplementary Table S4 online). PCA performed by combining ERM and IRM expression datasets indicated that the variance among maize lines was mostly explained for Oh40B and Mo17 by *RiPT*s expression in IRM and ERM, while B73, and two replicates out of three of Oh43, were located in the opposite quadrants of the biplot (see Supplementary Fig. S1 online).

### Phenotypic traits of maize inbred lines and *R. irregulare* mycorrhizal networks

Mycorrhizal plants belonging to the four inbred lines did not show significant differences in colonization levels, percentages of arbuscules, vesicles, hyphae, root length and shoot dry biomass (Table 1, Supplementary Table S5 online). However, B73 and Oh43 displayed the largest increments in mycorrhizal plants shoot P content compared with the other two lines, which are less dependent on mycorrhizal symbiosis (Mo17 and Oh40B) (Table 1).

**Table 1:** Phenotypic traits (mean ± standard error of the mean) of *Zea mays* plants belonging to four inbred lines, grown in symbiosis with *Rhizoglomus irregulare* under low phosphorus (P) availability (0.47 mg kg^−1^).

	Mycorrhizal root length (%)^z^	Total root length (cm)	Shoot dry weight (mg)	Shoot P (%)^z^	Shoot P content (mg)	Mycorrhizal shoot P increment (%) ^x z^
B73	57.7 ± 2.7 a	624.3 ± 171.1 a	344.3 ± 114.3 a	0.042 ± 0.013 b	0.13 ± 0.03 b	12.9 ± 4.4 a
Mo17	48.7 ± 4.7 a	452.3 ± 50.0 a	259.0 ± 29.7 a	0.14 ± 0.037 a	0.34 ± 0.05 a	0.9 ± 0.5 b
Oh40B	51.6 ± 2.0 a	688.0 ± 127.0 a	378.0 ± 7.5 a	0.057 ± 0.003 ab	0.22 ± 0.09 ab	1.0 ± 0.1 b
Oh43	48.3 ± 5.8 a	856.0 ± 297.2 a	280.0 ± 61.0 a	0.042 ± 0.008 b	0.12 ± 0.04 b	26.8 ± 5.5 a
ANOVA F_3,8_	1.11	0.82		6.68	8.01	22.44
Welch’s			4.18			
*P*	0.4	0.52	0.11	0.01	0.01	< 0.001

For each trait, F and *P* values resulting from one-way ANOVA, or Welch’s test for unequal variances, are reported. In columns, values followed by the same letter do not differ significantly at *P* ≤ 0.05 by Tukey’s HSD or Tamhane T2 test.

^z^ statistics performed on arcsine square-root-transformed data. ^x^ calculated as: (Mycorrhizal plant shoot P content − (mean control P content)/(mean control P content).

**Figure 5:**
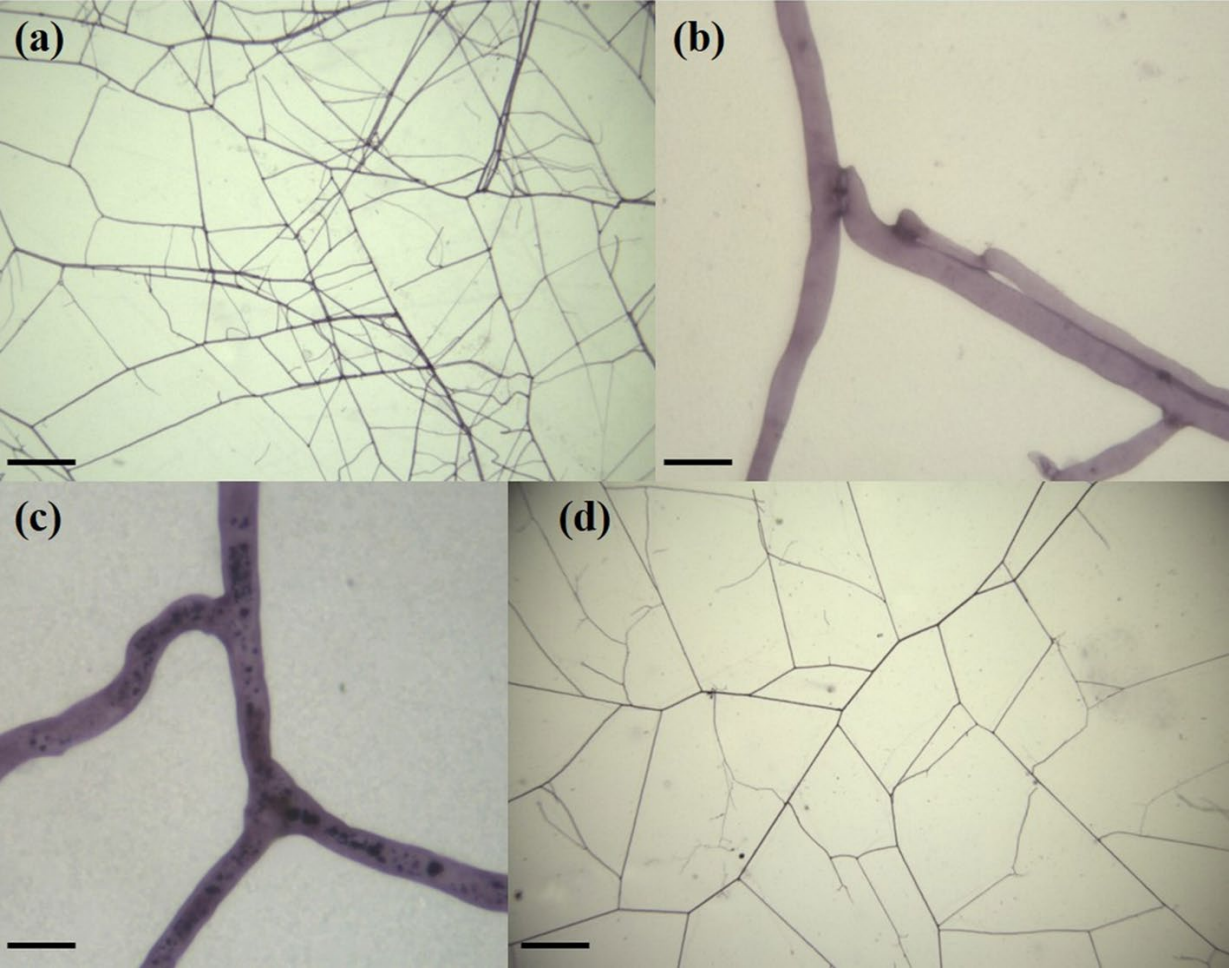
Membranes bearing *Rhizoglomus irregulare* IMA6 extraradical mycelium (ERM) produced in symbiosis with *Zea mays*, after Trypan blue staining (a, b, d) or succinate dehydrogenase (SDH) activity localization (**c**). (**a**) Highly interconnected ERM produced in symbiosis with maize line B73, scale bar = 160 μm; (**b**) multiple hyphal anastomoses in ERM growing from B73 roots, scale bar = 17 μm; (**c**) SDH-positive (viable) hyphal anastomoses, indicated by the deposition of blue formazan salts, in ERM produced in symbiosis with maize line Mo17, scale bar = 12.5 μm; (**d**) ERM growing from roots of the maize line Mo17, scale bar = 170 μm.

ERM growing from *R. irregulare*-colonized roots of the inbred line B73 showed the highest hyphal density, consistently with its branching density (Fig. 5), while ERM originating from roots of the line Mo17 was characterized by the lowest values, displaying significantly reduced hyphal (− 43.6%) and branching (− 42.9%) densities, compared with those of B73 (Table 2). ERM developing from Mo17 maize plants showed the highest values of hyphal exploration capacity.

**Table 2:** Phenotypic traits (mean ± standard error of the mean) of extraradical mycelium (ERM) formed by *Rhizoglomus irregulare* in symbiosis with four *Zea mays* inbred lines, under low phosphorus (P) availability (0.47 mg kg^−1^).

	Hyphal density (mm mm^−2^)	Explored area (cm^2^)	Length (m)	ERM anastomosis density (anastomoses mm hypha^−1^)	ERM branch density (branches mm hypha^−1^)
B73	10.1 ± 1.0 a	118.0 ± 3.2 ab	120.1 ± 14.1 a	0.74 ± 0.06 a	0.90 ± 0.08 a
Mo17	5.7 ± 0.7 b	132.3 ± 11.0 a	76.8 ± 15.5 a	0.81 ± 0.04 a	0.46 ± 0.02 b
Oh40B	8.9 ± 0.8 ab	118.3 ± 4.8 ab	106.5 ± 13.1 a	0.66 ± 0.04 a	0.82 ± 0.15 ab
Oh43	9.2 ± 0.6 ab	91.6 ± 5.4 b	84.9 ± 9.0 a	0.38 ± 0.05 b	0.68 ± 0.03 ab
ANOVA F_3,8_	6.11	6.31	2.27		
Welch’s				9.22	5.18
*P*	0.018	0.017	0.157	0.005	0.03

For each trait, F and *P* values resulting from one-way ANOVA, or Welch’s test for unequal variances, are reported. In columns, values followed by the same letter do not differ significantly at *P* ≤ 0.05 by Tukey’s HSD or Tamhane T2 test.

Shoot dry weight and its increases in mycorrhizal plants, compared with controls, were significantly correlated with ERM hyphal density (r = 0.58 and 0.59, respectively, *P* = 0.04) and with ERM length (r = 0.63, *P* = 0.03, and r = 0.70, *P* = 0.01, respectively). Moreover, in mycorrhizal maize the increase in shoot P content, with respect to controls, was negatively correlated with the ERM explored area, while P percentages showed a negative relationship with ERM density (see Supplementary Fig. S2 online). Accordingly, Mantel’s test computed to assess the relationship among data of the whole set of ERM structural traits and mycorrhizal plant benefit traits (increments in shoot dry weight and P content, in comparison with control plants) showed a significant correlation (r = 0.33, *P* = 0.0079).

### Correlation of *RiPT* and *ZmPht1* transcripts with plant/fungal phenotypic traits

**Figure 6:**
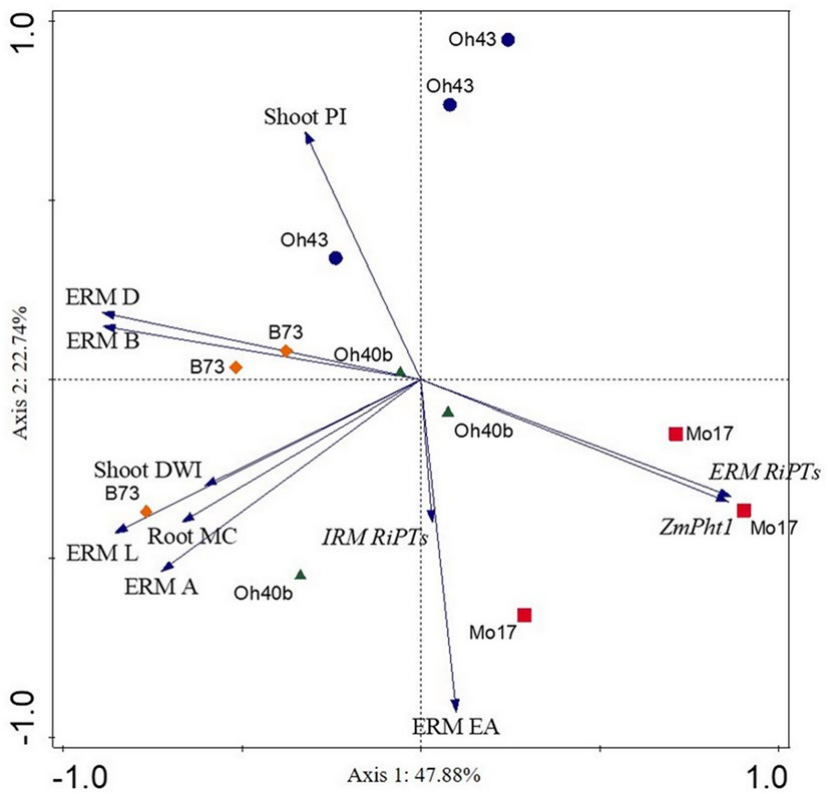
Principal Component Analysis (PCA) biplot showing the relations between physiological and molecular data collected from four *Zea mays* inbred lines grown under low phosphorus availability (0.47 mg kg^−1^) in symbiosis with *Rhizoglomus irregulare*. Standardized data of *R. irregulare* extraradical mycelial traits—mycelium density (ERM D, mm mm^−2^), length (ERM L, m), explored area (ERM EA, mm^2^), branching density (ERM B, branch mm^−2^) and interconnectedness (ERM A, anastomoses mm^−2^)—mycorrhizal *Zea mays* traits—shoot P increment over control plants (Shoot PI), shoot dry weight increment over controls (Shoot DWI) and mycorrhizal colonization percentage (Root MC)—and average relative expression levels of P transporters in mycorrhizal *Z. mays* roots (*ZmPht1*), in *R. irregulare* extraradical (ERM *RiPT*) and intraradical (IRM *RiPT*) mycelium were used. The first and second axes explain 70.62% of total variance.

Results of the PCA performed on the averages of each relative expression dataset (*ZmPht1*s, IRM and ERM *RiPT*s) of mycorrhizal plants, on ERM phenotypic traits measurements (explored area, hyphal density, length, branching and anastomosis densities) and on mycorrhizal maize shoot dry weight and P increments, explained 70.6% of the total variance (PC1 and PC2), and showed differential responses of the four maize inbred lines (Fig. 6). The fraction of variation in ERM phenotypic variables explained by the two axes in the PCA was high, with 52% to 79% of variance explained by ERM anastomosis, branching, hyphal densities and length, on the first axis, and 86% of variance explained by ERM explored area on the second axis. Among plant phenotypic traits, only 11% of variation in shoot P increments was explained by the first, and 48% by the second axis, whereas 37% and 45% of variation in shoot dry weight increments and mycorrhizal colonization of roots, respectively, was explained by the first axis. Analysis of expression data indicated that variation in maize and ERM *PT*s explained by the first axis represented 74% and 75%, respectively, while IRM *PT*s showed a low contribution, 16% only, on the second axis. The separation of Mo17 samples was mostly explained by high levels of both plant and fungal P transporters expression (Fig. 6), as in the PCA performed on separate datasets of expression data (Fig. 3 and Supplementary Fig. S1 online). On the contrary, high values of some ERM and plant phenotypic traits (anastomosis/branch densities and mycorrhizal colonization/shoot growth) better explained separation of data from B73 line in the PCA. While the maize line Oh40B variation was not clearly explained by any of the traits studied, PCA suggested that P increases in shoots of mycorrhizal plants explained Oh43 samples separation, mostly along the second axis (Fig. 6).

The analyses also suggested that ERM *RiPT*s and root *ZmPht1s* expression were highly correlated, as confirmed by a significant Mantel’s test (r = 0.69, *P* = 0.001). Mantel’s tests did not show significant correlation among data of IRM *RiPT*s expression with those of *ZmPht1* or ERM *RiPT*s (both *P* > 0.6). Mantel’s test computed to assess the relationship among data of ERM phenotypic traits and AMF/maize *PT*s expression levels showed significant correlations for both datasets (r = 0.30, *P* = 0.032 and r = 0.55, *P* = 0.0004, for ERM *RiPT*s and *ZmPht1*s, respectively).

## Discussion

This study showed that, at low-P availability, the four assayed mycorrhizal maize inbred lines (B73, Mo17, Oh40B, Oh43) differed in their fungal/plant *PTs* expression patterns, in plant/ERM phenotypic traits, and in the relationships between molecular/phenotypic features. Interestingly, mycorrhizal plants of the low-P tolerant (and poorly-mycorrhizal responsive) maize line Mo17 displayed increased expression of roots and ERM phosphate transporters, compared with the low-P susceptible (and highly-mycorrhizal responsive) line B73 which revealed larger ERM hyphal and branch densities.

The inbred lines assayed here, when grown in the absence of mycorrhizal symbiosis, were characterized by differential responses to low-P status. Previous works reported that some *ZmPht1* genes were induced by P starvation, although findings were not consistent among studies carried out on different plant species^25,32^ and experimental systems^38,41–46^. Here, high expression values of *ZmPht1;1*, *ZmPht1;3*, *ZmPht1;9*, *ZmPht1;11* and *ZmPht1;12* were found in control maize lines, suggesting that plants were responding to the low-P availability. Actually, the molecular mechanisms of P acquisition and distribution are still unclear in maize and the results of *ZmPht1* genes expression in genotypes differing for their low-P tolerance are often inconsistent: in this work the low-P tolerant maize line Mo17 did not show higher *ZmPht1* genes transcription, as induced by P deficiency, compared with the low-P susceptible line B73. Accordingly, no differential expression of *ZmPht1;1*, *ZmPht1;2* and *ZmPht1;4* was found among maize lines differing for their low-P tolerance^42^, while even downregulation of *ZmPht1;1* and *ZmPht1;2* was detected in response to P deprivation^47^. Besides the activity of this transporter family, further mechanisms adopted by plants to facilitate P uptake may play a major role in the response of low-P tolerant maize lines. Indeed, upregulation of genes, whose transcripts abundance modulates P allocation between above- and belowground tissues, as reported for *ZmCCD10*, may facilitate P flow to shoots, thus inducing roots to absorb and/or translocate P^47^. Moreover, some low-P tolerant maize lines showed variations in root morphology (increased topsoil foraging, root hairs and lateral roots) that may explain differential maize responses to low-P availability^42^.

Here, most *ZmPht1* genes were downregulated in mycorrhizal B73 roots and upregulated in mycorrhizal Mo17 line. Upregulation of *ZmPht1;4*, *ZmPht1;5* and *ZmPht1;8* genes, which were previously suggested to be induced by either P starvation or mycorrhizal colonization^27,38,43^, was detected in mycorrhizal Mo17 line. In this work the expression of *ZmPht1;1* and *ZmPht1;3* genes was downregulated in all the mycorrhizal inbred lines tested, consistently with previous findings on other maize genotypes^41,48,49^.

Phosphorus content of Mo17 control plants was very high, compared with the other lines, showing its ability to acquire P even at low-P levels, and not to benefit from mycorrhizas in terms of plant growth and P increases. This finding confirms also previous studies on mycorrhizal response variability among genotypes, with little mycorrhizal benefits in host lines capable of low-P tolerance^42,50^. Surprisingly, mycorrhizal Mo17 plants showed larger expression of 5 out of 8 *ZmPht1*s, compared with lines B73 and Oh43, both displaying high mycorrhizal P increases. Such results are difficult to explain when considering only gene expression data, due to the wide variation in symbiotic upregulation of individual *ZmPht1* genes, depending on the host and AMF genotype and on experimental systems, and to the lack of information on how external P levels affect the transcriptional regulation of *ZmPht1* genes induced by both P-starvation and mycorrhizal symbiosis.

Maize is well known for its exceptional genome structural diversity, including copy number variants and presence/absence variants, and there is growing evidence for the role of structural variation in maize adaptation and phenotypic differences^51^. Whole-genome, array-based, comparative genomic hybridization revealed a level of structural diversity between the inbred lines B73 and Mo17 that is unprecedented among higher eukaryotes. Several hundred copy number variation sequences have been observed among the two genotypes, as well as several thousand presence/absence variation sequences that are present in B73 but not Mo17^52^. Moreover, de novo assembly, annotation and comparative analysis of diverse maize genomes revealed previously unknown variation in gene content, genome structure, and methylation^53^. Such high structural and epigenetic differences among maize lines may explain differences in P transport activity observed here. Nevertheless, present data on ERM characteristics may provide further insights into the contrasting results obtained from the different maize lines.

Actually, ERM growing from mycorrhizal roots of the four inbred lines showed large differences in its structural traits, such as hyphal density, branching density and interconnectedness. An overall correlation between ERM structural traits and both *ZmPht1*s and ERM *RiPT*s expression was found in mycorrhizal plants, suggesting that, independently on host genotype specific modulation of plant and AMF transporters, ERM characteristics play a fundamental role in symbiotic nutritional fluxes. Here, B73 and Mo17 mycorrhizal plants showed statistically similar biomass, that was associated in B73 line to larger ERM hyphal density and interconnectedness, lower plant P levels and higher mycorrhizal P increases, and in Mo17 line, to higher *ZmPht1*s expression levels, larger host P percentages and lower mycorrhizal P increases. Such divergent behaviors, characterized by the combination of different traits, suggested the occurrence of complex and different response strategies to low-P availability. Data similar to those obtained for B73 on shoot P increment, *ZmPht1*s/*RiPT*s transcript levels and ERM hyphal densities were obtained in Oh43 mycorrhizal plants, confirming the identification of such lines as among the best mycorrhiza-responsive ones in low-P conditions^32,54,55^. Interestingly, previous findings showed that Oh43 was highly responsive to *R. irregulare* in terms of P uptake, and that this responsiveness was better explained by ERM abundance than mycorrhizal root colonization or *ZmPht1* transcripts level^38^. In our experimental system, ERM produced by *R. irregulare* in symbiosis with Oh43 line did not show the largest values in the assessed phenotypic variables or in *RiPT *expression, compared with the other lines. Such findings may be ascribed to the variability of experimental systems, sampling plans and monitoring endpoints adopted to characterize ERM traits and investigate the role of ERM in host P acquisition^56–59^.

Here, ERM structural traits showed also a significant overall correlation with mycorrhizal host benefit traits (increases in growth and P content). Previous studies correlated the abundance of extraradical hyphae with the efficiency of fungal P uptake^56,60^ and a recent one confirmed the relationships between extraradical hyphal length and the contribution of mycorrhizal phosphate uptake pathway to maize P acquisition^38,61^. These relationships are worth investigating further, in order to disclose all ERM structural variables potentially relevant to the analysis of host/fungal P acquisition mechanisms. Nevertheless, they also confirm that fungal genotypes and multiple traits, both structural and functional, are differentially involved in the regulation of P foraging capacity and translocation in AMF networks. As plant P acquisition derives from the modulation of mycorrhizal and plant P acquisition pathways, the role of ERM abundance in plant P nutrition is strictly dependent on the assortment of plant/AMF genotypes and on the availability of soil P to plants. Differences detected in *R. irregulare* PT expression in symbiosis with different maize lines suggest that host genotypes may provide unequal lipid/sugars fluxes to the fungus, and in turn require diverse levels of mineral nutrients from the mycorrhiza-dependent pathway, probably affecting ERM growth and the foraging reward by the symbiotic fungus.

Our findings suggest that functional variables relevant to mycelial networks properties may be influenced by fungal foraging strategies, namely interconnectedness, nutrient transport, construction cost, and resilience, which have been studied in different fungal species^62^. Accordingly, the high interconnectedness, functional to multidirectional transport, of the fungal network formed by *R. irregulare* with B73 plants, which are less tolerant to P starvation, may have facilitated P exploitation of resources over exploration, allowing the host to acquire a very high proportion of its P from the fungus, downregulating most of its *ZmPht1* genes. On the contrary, the less interconnected hyphal network growing from Mo17 roots suggests a stronger role in exploration than in resources exploitation, as the plant maintained high P levels in its tissues by upregulating most of its *ZmPht1* genes.

Further studies should assess the genetic basis of mycorrhizal host responses, by coupling the quantification of phenotypic/functional traits in diverse maize genotypes with quantitative trait loci mapping, as previously reported for mycorrhizal colonization in wheat^36,37^. Findings from such studies would boost new targeted molecular breeding strategies and the improvement of our knowledge on the genetic mechanisms regulating P acquisition and ERM development and functioning.

## Methods

### Experimental set-up and growth conditions

Twenty seeds for each maize inbred line were germinated in sterile conditions in the dark at 25 °C after surface sterilization in 96% ethanol for 1 min, 3.5% sodium hypochlorite solution for 30 min, followed by several washing steps with sterile water. After 7–10 days, a half of maize plantlets for each line were inoculated with *Rhizoglomus irregulare* (Błaszk., Wubet, Renker & Buscot) Sieverd., G.A. Silva & Oehl (University of Pisa, isolate IMA6). The inoculum was obtained from *Medicago sativa* pot-culture soil after wet sieving through a 100-μm-mesh size sieve. Each root system was wrapped in a nylon net (41 μm mesh) in order to obtain a flat mesh pocket limiting root growth in the volume inside the nylon net (about 65 cm^3^). Wrapped inoculated plantlets and non-inoculated controls were transferred into perforated 14 cm Petri dishes, allowing the growth of maize shoots outside the system. The root systems were covered with sterile grit and moistened with 10 ml distilled water. After sealing with Parafilm, the Petri dishes were wrapped with aluminum foil, placed into sun-transparent bags and maintained in a growth chamber at 25 °C, with 16 h of light per day. The plants were watered weekly with 3 ml of modified Hoagland solution containing ½ strength of the standard concentration of KH_2_PO_4_.

After 7 weeks’ growth the nylon net was removed and roots were washed with distilled water, placed between two 13 cm diameter membranes (cellulose esters, 0.45 μm pore diameter size) to allow the growth of extraradical mycelial networks^63^, which were placed into new perforated Petri dishes containing sterile quartz grit and treated as described above. Mycorrhizal and control plants were maintained in the latter system and watered weekly with 3 ml of the modified Hoagland solution described above. On average, during the experiment 37.5 µM KH_2_PO_4_ were supplied to each plant in the growth solution.

The root sandwich systems were opened after 3 weeks’ growth (see Supplementary Fig. S3, S4 online), three biological replicates per each line were used for molecular analyses and three further biological replicates were used for ERM structural traits analyses, IRM assessment, total and mycorrhizal root length, shoot biomass production and shoot P content. Plant tissues P percentages were measured after sulphuric/perchloric acid digestion using the photometric method^64^.

### Harvest and total RNA extraction

The ERM spreading from the roots and growing on the surfaces of the membranes was harvested on ice-cold sterile distilled water using two forceps under a dissecting microscope. Colonized roots were selected by visualizing autofluorescence of arbuscules under blue light, using an inverted fluorescence microscope, cut from the rest of the plant and collected on ice. Collected ERM and colonized roots, after removing excess water with a filter paper, were immediately weighed, and transferred in liquid nitrogen. Different aliquots of ERM (1.5 to 5 mg of fresh biomass) and of colonized and control roots (100 mg of fresh biomass) per each biological replicate were collected and stored at − 80 °C until RNA extraction. RNA from ERM was extracted using the MasterPure Complete DNA and RNA Purification Kit (Epicenter), according to the manufacturer’s instructions and eluted in TE with RiboGuard 40 U/µl. RNA from colonized and control roots was extracted using NucleoSpin RNA Plus kit (Macherey–Nagel) according to the manufacturer’s instructions and eluted in RNase-free water. RNA was quantified with the Qubit RNA HS Assay kit and stored at − 80 °C.

### Quantitative gene expression analysis of *Z. mays* P transporter (*ZmPht1*) genes in maize roots

RNA extracted from colonized and non-colonized (control) maize roots was used for gene expression analysis of the *Z. mays* P transporters (*ZmPht1*). RNA extracts were DNase treated and cDNA from three biological replicates for each line was synthesized using High-Capacity cDNA Reverse Transcription Kits (Applied Biosystems, Italy), according to the manufacturer’s protocol. The synthesized cDNA was quantified with the Qubit RNA HS Assay kit and stored at − 20 °C. The PCR program was conducted in an Eppendorf Mastercycler thermal cycler according to these parameters: 95 °C for 3 min and then 35 cycles of 95 °C for 40 s, 57 °C for 40 s and 72 °C for 40 s. PCR reactions were performed in a final volume of 15 µl, containing 10 × GoTaq Reaction Buffer, 100 × BSA, 50 mM MgCl_2_, 10 µM of dNTPs, 10 μM of each primer, 5 unit of GoTaq DNA Polymerase, and 5 ng of cDNA. The PCR products were checked on 2.5% TBE agarose gel and visualized by EuroSafe staining.

The qRT-PCR was carried out in a final volume of 25 μl containing 12.5 μl of iQ SYBR Green Supermix 2 × (Bio-Rad, Italy), 2 μM of each primer and 5 ng cDNA template using a CFX Connect real-time PCR Detection System (Bio-Rad). The qRT-PCR program was used as follows: 95 °C for 10 min, followed by 40 cycles at 95 °C for 15 s and 60 °C for 1 min and to calibrate gene expression data, a maize housekeeping gene *ZmActin1*^43^, was used as an internal control. The relative transcript level was calculated as 2^−ΔΔCT^ and the reactions were performed on three biological samples with two technical replicates. The specificity of qRT-PCR amplification was assessed using a melting curve analysis of 60 to 95 °C after the final PCR cycle^65^. The primers used were those reported by Liu et al.^43^.

### Quantitative gene expression analysis of *R. irregulare* P transporter (*RiPT*) genes in ERM/IRM

To remove genomic DNA traces, the ERM and root RNA samples were treated with DNase, according to the manufacturer’s instructions. cDNAs (100 ng each sample) were synthesized using the iScript Select cDNA Synthesis kit (Bio-Rad) and stored at − 20 °C. For *R. irregulare* P transporters (*RiPTs*) gene expression analysis, the following primers were used: RiPT1, RiPT3, RiPT5 and RiPT7^39^, while the pair RiPT2 F (5′-TTGGTGTCCTTACTGCACTC-3′) and RiPT2 R (5′- TGTTGTTGTGTTTGGACCGA-3′), was designed in Primer 3 (http://frodo.wi.mit.edu/cgi-bin/primer3/primer3_www.cgi) and tested in Amplify 3.1 (http://engels.genetics.wisc.edu/amplify) based on the partial RNA sequence of the *R. irregulare* phosphate transporter 2 (*RiPT2*)^66^.

Quantitative real time polymerase chain reaction (qRT-PCR) was performed using a CFX Connect real-time PCR Detection System. Reactions were carried out in a final volume of 20 μl containing 10 μl of iQ SYBR Green Supermix 2 × , 0.2 μM of each primer and 5 ng cDNA template. The PCR thermal program consisted in an initial incubation at 95 °C for 3 min, followed by 40 cycles of 95 °C for 30 s, 60 °C for 1 min and 72 °C for 30 s, where the fluorescence signal was measured^21^. At the end of each cycle, a melting curve was recorded (from 60 to 95 °C), to assess the specificity of the amplification product and therefore to exclude the formation of non-specific products by the primers^65^. The efficiency of the primers was tested by performing qRT-PCR on different cDNA dilutions. All reactions were performed at least two times on three biological and two technical replicates. All gene expression data were normalized to the expression of the *elongation factor 1-α* (*ef1-α*) using primers ef1-α F and ef1-α^57^. The relative levels of transporters transcription were calculated by using the 2^−ΔCT^ method (ratio (reference/target) = 2^CT(reference) − CT(target)^), a variation of the 2^−ΔΔCT^ method^67^.

### Phenotypic traits of maize inbred lines and *R. irregulare* mycorrhizal networks

Three biological replicates for each maize line were used to measure shoot P percentages and shoot biomass of plants grown in the sandwich systems and to determine AMF root colonization and ERM structural traits. The shoot of each plant was cut and oven-dried at 50 °C until constant weight. Roots and ERM growing on the membranes were stained for the localization of succinate dehydrogenase (SDH) activity. After the SDH test, the same membranes were stained with Trypan blue in lactic acid (0.05%). These staining methods allowed the assessment of mean ERM density (expressed as mm of hyphal length per mm^2^ of membrane surface) and the area explored by ERM (expressed in mm^2^), under the dissecting microscope^68^. For each membrane, mean ERM density was measured with a grid eyepiece by evaluating the length of hyphae in 15 areas of 9.8 mm^2^, using the gridline intersect method, while the area explored by ERM was estimated with a transparent 5-mm-square grid. Total ERM length was calculated as the product of mean ERM density and area obtained from each replicate membrane. For each treatment and replicate membrane, five regions (32 × 20 mm each) covered by ERM were cut, mounted on microscope slides and examined under a Reichert-Jung Polyvar microscope to assess the number of hyphal contacts, anastomoses and branches^68^, which were referred to the hyphal length and to the observed area.

Mycorrhizal colonization was evaluated by clearing the roots with 10% KOH in a water bath at 80 °C for 15 min and subsequently staining them with Trypan blue in lactic acid (0.05%) after a treatment in 2% HCl for 10 min. Mycorrhizal and total root length, along with percentage of arbuscules, vesicles and hyphae, were estimated under a dissecting microscope using the grid line intersect method^69^.

### Data analysis

Data of fungal and plant P transporter expression and data of mycelial growth and viability were logarithmically transformed, when needed, to fulfil ANOVA assumptions and subjected to one-way ANOVA with inbred line as the source of variation, followed by Tukey’s HSD post hoc test. Welch’s test for Unequal Variances, followed by Tamhane's post hoc test, was used when the variance homogeneity assumption was not satisfied. A probability level of *P* < 0.05 was considered for all tests. Percentage data were analyzed after arcsine transformation. Gene expression data analysed in this work are reported in Supplementary Tables S5 and S6 online. Multivariate (Mantel’s test, permutations 9999) and linear regression analyses were performed to determine the correlations among gene expression data and between them and phenotypic traits of both plants and ERM. Principal Component Analyses (PCA) were computed to assess the relationships between physiological and molecular data collected from four *Z. mays* inbred lines in symbiosis or not with *R. irregulare*. Statistical analyses and graphics were generated using the software tools R (package pheatmap), SPSS (version 23), PAST (version 4.03) and CANOCO (version 5.0).

### Additional statements

The maize genotypes employed in this study are public lines. They were obtained from the Maize Genetics Cooperation Stock Center, U.S. National Plant Germplasm System (https://www.maizegdb.org/data_center/stock) and maintained by sibling at the Department of Sustainable Crop Production, Piacenza, Italy. All the experimental research studies on plants carried out in this work, including the collection of plant material, are complied with relevant institutional, national, and international guidelines and legislation.

## Supplementary Information

Supplementary Information 1.

Supplementary Tables.

## Data Availability

All data generated or analysed during this study are included in this published article (and its Supplementary Information files).
