# Supplementary material for: Diverse mycorrhizal maize inbred lines differentially modulate mycelial traits and the expression of plant and fungal phosphate transporters
Source: Sci Rep. 2022 Dec 8;12:21279. doi: 10.1038/s41598-022-25834-7 (PMC9732053; doi:10.1038/s41598-022-25834-7)
Supplement: Supplementary file 1 — Supplementary Information 1. [file 41598_2022_25834_MOESM1_ESM.docx]

**Supplementary Information**

**Scientific Reports**

**Article title:** Diverse mycorrhizal maize inbred lines differentially modulate mycelial traits and the expression of plant and fungal phosphate transporters

**Authors:** Luca Giovannini, Cristiana Sbrana, Manuela Giovannetti, Luciano Avio, Alessandra Lanubile, Adriano Marocco, Alessandra Turrini

Corresponding author:

Alessandra Turrini, Department of Agriculture, Food and Environment, University of Pisa, Via del Borghetto 80, 56124, Phone: +39 0502216646, Fax: +39 0502210606, e-mail address: [alessandra.turrini@unipi.it](mailto:alessandra.turrini@unipi.it)

**Fig. S1.** Principal Components Analysis (PCA) biplot of the relative abundance of P transporters transcripts in *Rhizoglomus irregulare* extraradical (ERM *PT*s) and intraradical (IRM *PT*s) mycelium produced in symbiosis with four *Zea mays* inbred lines grown under low phosphorus availability (0.47 mg kg^-1^). The first and second axes explain 51.37% of total variance.


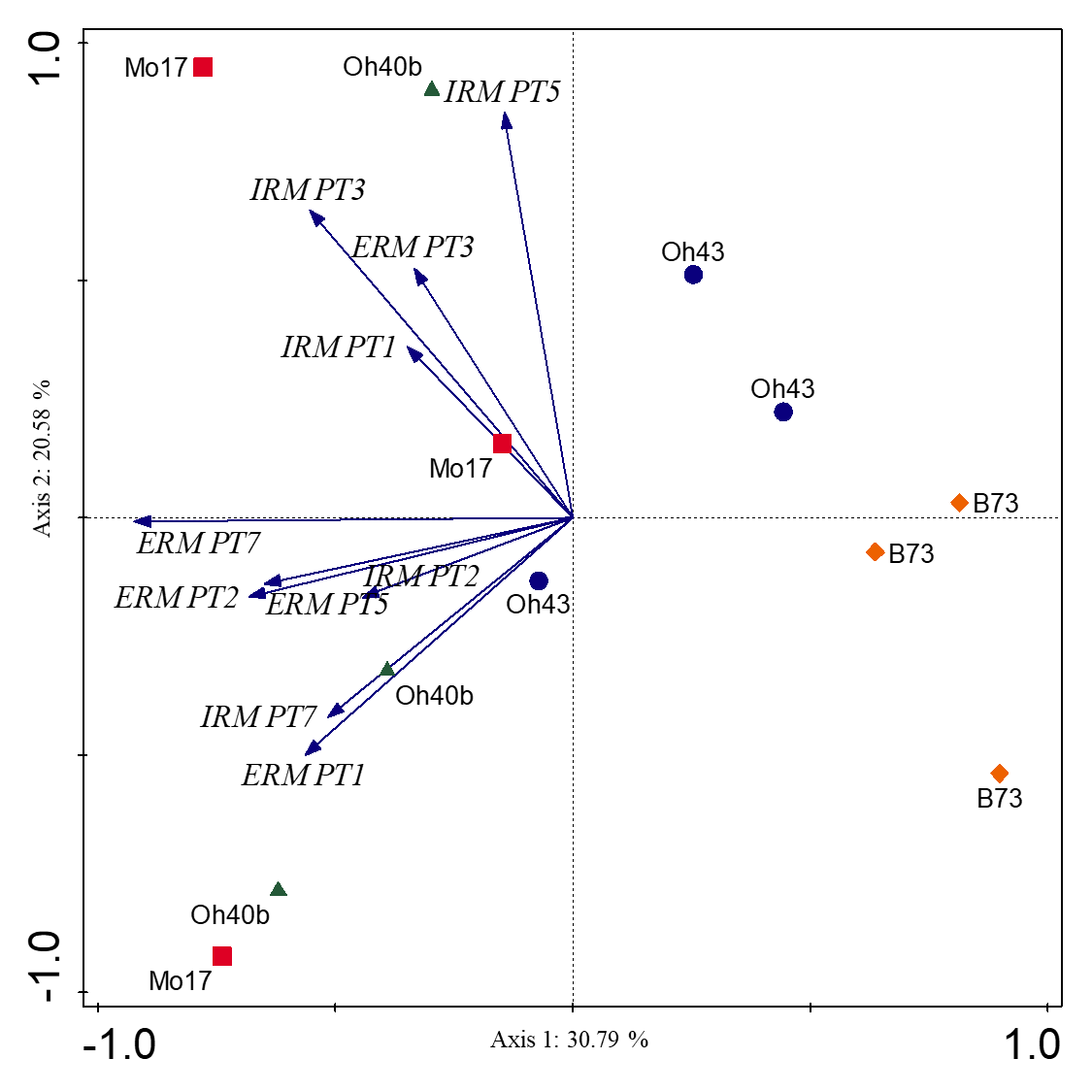


**Fig. S2.** Plots showing linear regression curves obtained from phenotypic data of *Zea mays* inbred lines in symbiosis with *Rhizoglomus irregulare*. Curves represent 95% confidence intervals. In (a), R = -0.63 P = 0.03; in (b), R = -0.78 P = 0.003. Shoot P increment (%) was calculated as (mycorrhizal plant shoot P content – mean control P content)/mean control P content.


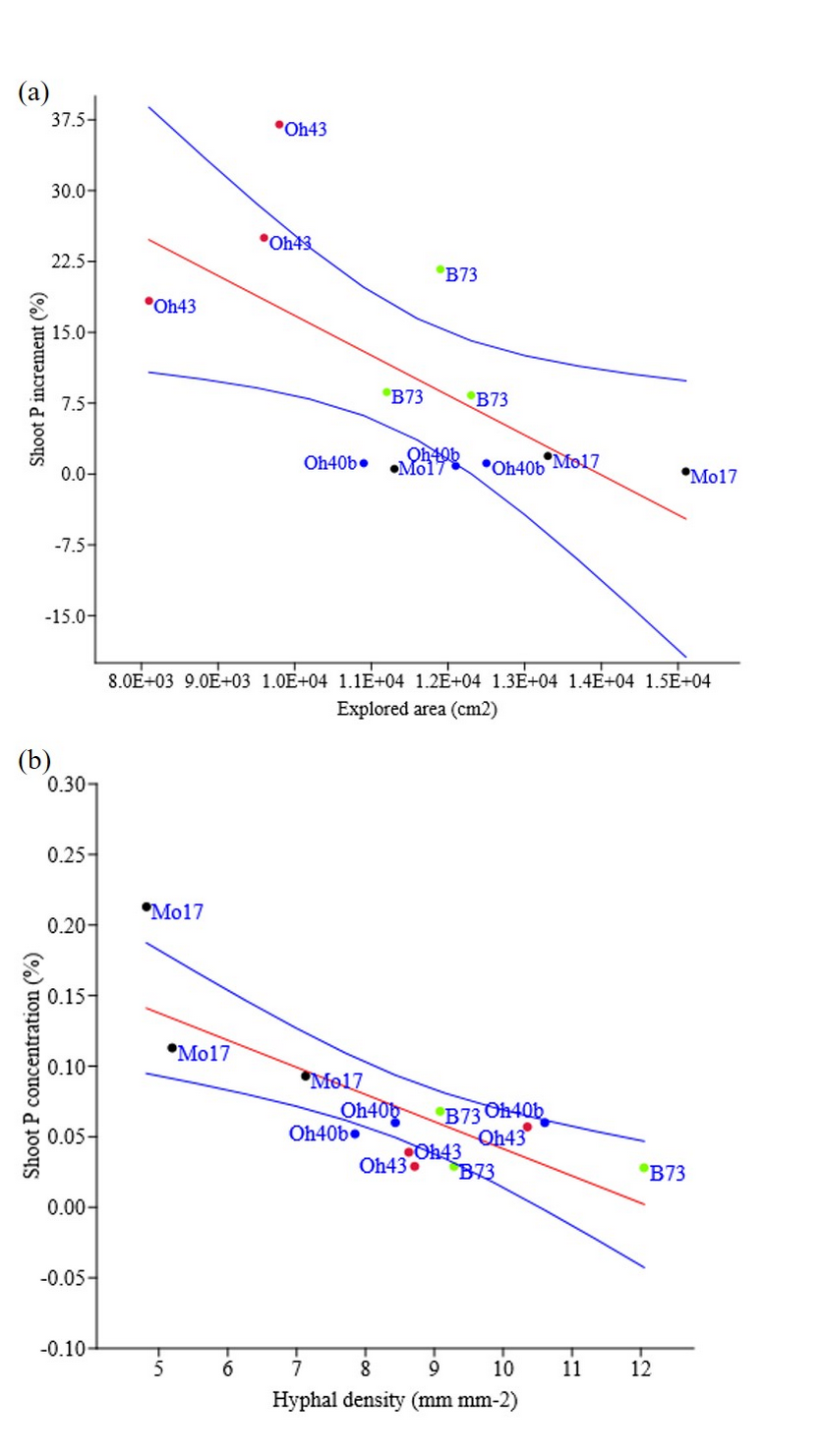


**Fig. S3.** Different stages of maize growth in the experimental system devised to analyse molecular and structural plant and fungal traits. (a) Maize plant inserted in perforated 14 cm Petri dishes at the beginning of mycorrhizal colonization stage. (b) Maize plants grown for seven weeks in the experimental system, placed in sun-transparent bags. (c) Maize roots and *Rhizoglomus irregulare* extraradical mycelium spreading from roots on 13 cm diameter cellulose esters membranes at the end of the experiment.


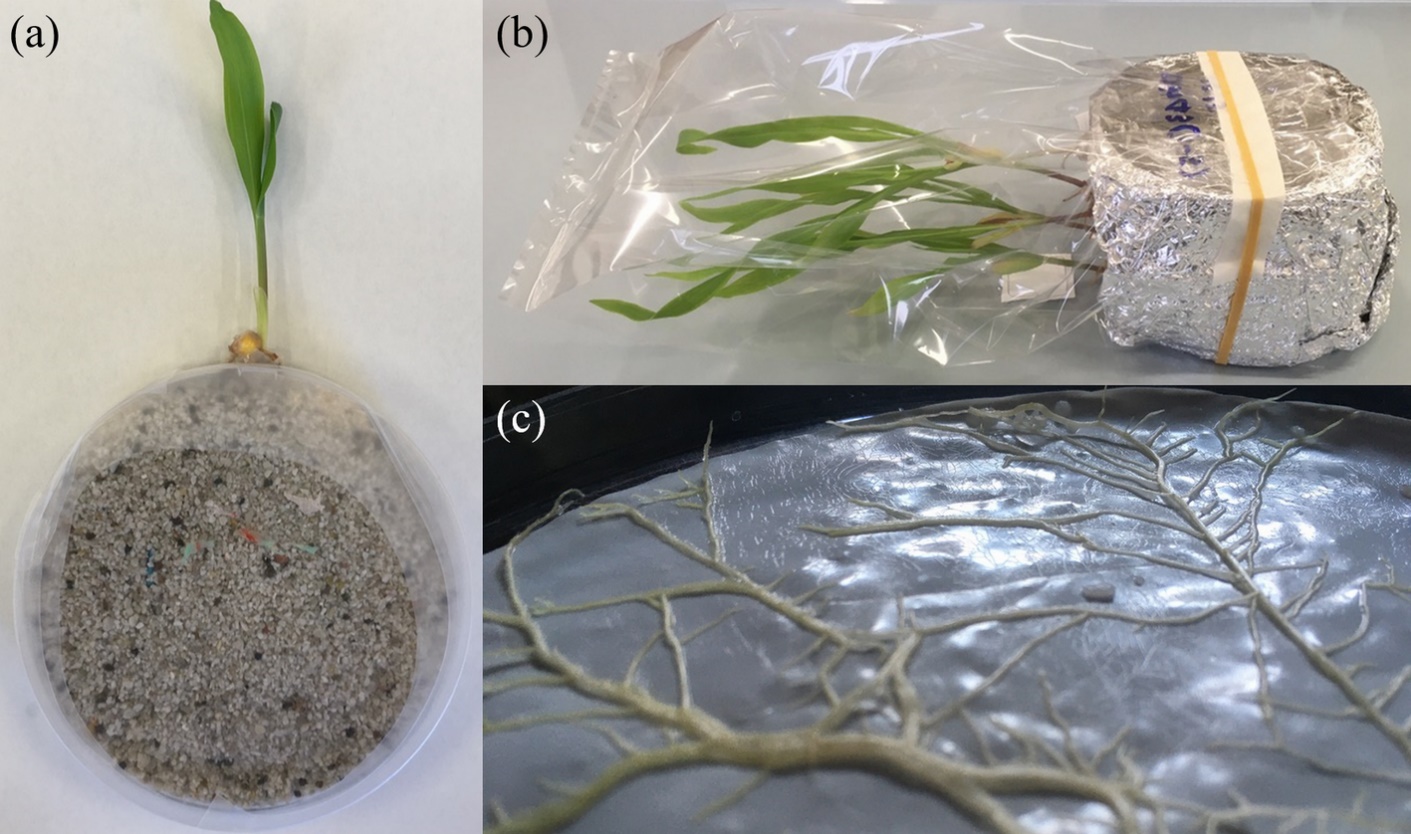


**Fig. S4.** *In vivo* production of extraradical mycelium and roots containing intraradical fungal structures of *Rhizoglomus irregulare* in symbiosis with *Zea mays* inbred lines, and their collection for molecular analyses.


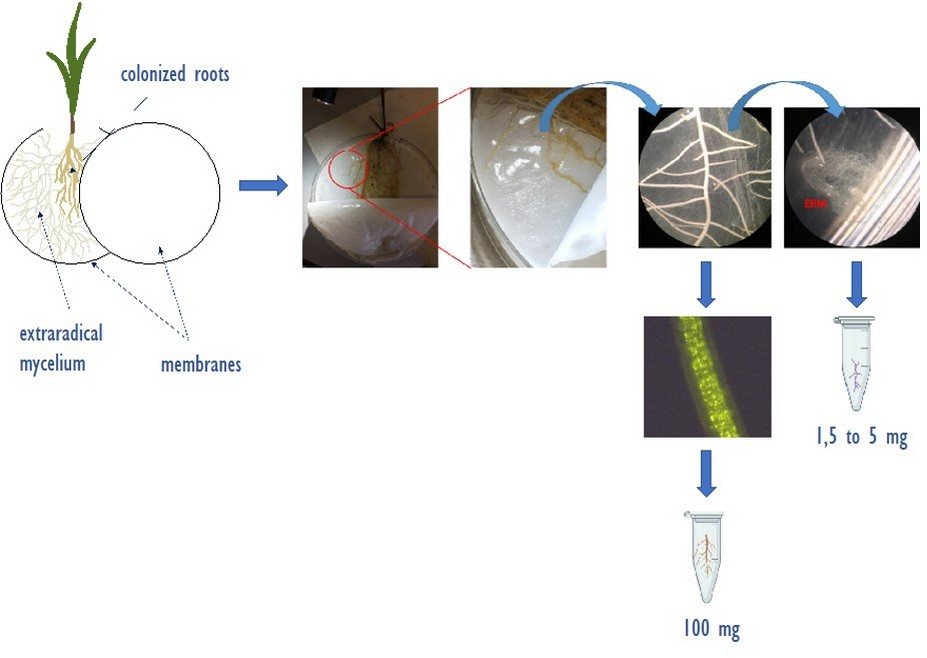


**Table S1.** Normalized values (mean ± standard error of the mean, the displayed values are multiplied by 10^2^) of Zea mays *Pht1* gene expression in roots of plants grown in symbiosis (Mycorrhizal) or not (Control) with the arbuscular mycorrhizal fungus *Rhizoglomus irregulare* under low phosphorus availability (0.47 mg kg^-1^). Transcripts were normalized against the *ZmActin1* gene. For each *Pht1* gene, F and P values from one way ANOVA, or Welch’s test for unequal variances, among maize lines are given at the end of the table. In columns, values followed by the same letter (a-c for controls, x-y for mycorrhizal plants) do not differ significantly at P ≤ 0.05 by Tukey’s HSD or Tamhane T2 test.

| **Maize line** | **Treatment** | ***ZmPht1;1*** | ***ZmPht1;3*** | ***ZmPht1;4*** | ***ZmPht1;5*** | ***ZmPht1;8*** | ***ZmPht1;9*** | ***ZmPht1;11*** | ***ZmPht1;12*** |
| --- | --- | --- | --- | --- | --- | --- | --- | --- | --- |
| **B73** | **Control** | 4.1 ± 0.9 | 11.1 ± 2.8 ab | 0.5 ± 0.3 ab | 2 ± 0.6 | 0.7 ± 0.2 a | 16.2 ± 3.9 b | 18.4 ± 2.7a | 30.2 ± 4.3a |
|  | **Mycorrhizal** | 2.4 ± 1.7 | 1.3 ± 0.9 xy | 0.5 ± 0.2 y | 1.3 ± 1.7 | 0.2 ± 0.01 y | 12.6 ± 9.8 | 3 ± 3.7 y | 1.9 ± 1.4 |
|  | **F_1,4_** | 2.29 | 32.58 | 0.02 | 0.39 | 10.41 | 0.35 | 33.88 | 118.49 |
|  | **P** | 0.20 | **0.005** | 0.89 | 0.57 | **0.03** | 0.59 | **0.004** | **<0.001** |
| **Mo17** | **Control** | 3.2 ± 1.5 | 1.8 ± 1.3 c | 0.06 ± 0.01 b | 0.2 ± 0.01 | 0.1 ± 0.01 b | 12.6 ± 6.8 b | 1.2 ± 0.3 b | 4.8 ± 4.4 b |
|  | **Mycorrhizal** | 2.7 ± 1.3 | 0.5 ± 0.5 y | 8.8 ± 1.1 x | 3.8 ± 0.1 | 1.2 ± 0.1 x | 11.6 ± 6.0 | 13.1 ± 2.8 x | 9.1 ± 4.0 |
|  | **F_1,4_** | 0.14 | 2.33 | 191.49 | 2786.08 | 455.08 | 0.04 | 55.10 | 1.52 |
|  | **P** | 0.72 | 0.20 | **<0.001** | **<0.001** | **<0.001** | 0.86 | **0.002** | 0.28 |
| **Oh40B** | **Control** | 6.1 ± 1.6 | 13.9 ± 0.8 a | 0.6 ± 0.3 ab | 0.8 ± 0.4 | 0.1 ± 0.09 b | 32.4 ± 4.8 a | 7.3 ± 3.3 ab | 13.6 ± 5.0 ab |
|  | **Mycorrhizal** | 3.2 ± 1.3 | 0.8 ± 0.01 x | 1.8 ± 0.3 y | 1.1 ± 0.2 | 0.3 ± 0.06 y | 17.3 ± 1.4 | 4.2 ± 2 xy | 3.7 ± 3.5 |
|  | **F_1,4_** | 5.80 | 869.79 | 19.62 | 2.02 | 10.92 | 27.26 | 2.01 | 7.78 |
|  | **P** | 0.07 | **<0.001** | **0.01** | 0.23 | **0.03** | **0.006** | 0.23 | **0.049** |
| **Oh43** | **Control** | 7.8 ± 4.3 | 7.7 ± 2.4 b | 0.2 ± 0.03 a | 1.6 ± 1.4 | 0.3 ± 0.1 b | 14.7 ± 4.8 b | 14.7 ± 11.7 ab | 21 ± 17.5 ab |
|  | **Mycorrhizal** | 7.1 ± 3.1 | 4.7 ± 4.0 xy | 7.1 ± 3.1 x | 2.6 ± 1.9 | 0.8 ± 0.3 x | 16.5 ± 6.9 | 8.1 ± 5.8 xy | 6.5 ± 6.4 |
|  | **F_1,4_** | 0.05 | 1.16 | 14.37 | 0.53 | 10.41 | 0.13 | 0.76 | 1.83 |
|  | **P** | 0.83 | 0.34 | **0.02** | 0.51 | **0.03** | 0.73 | 0.43 | 0.25 |
| **Control** | **ANOVA**  **F_3,8_**  **Welch’s**  **P** | 1.84  0.28 | **20.44**  **<0.001** | **21.88**  **0.009** | 8.48  0.047 | **10.84**  **0.003** | **9.09**  **0.006** | **31.32**  **0.006** | **13.49**  **0.012** |
| **Mycorrhizal** | **ANOVA**  **F_3,8_**  **Welch’s**  **P** | 3.62  0.06 | 0.99  0.49 | **17.46**  **0.001** | **83.59**  **0.001** | **27.56**  **<0.001** | 0.52  0.68 | **4.29**  **0.04** | 1.67  0.25 |

**Table S2.** Normalized relative expression values (mean ± standard error of the mean) of *Zea mays* *Pht1* gene in roots of plants grown in symbiosis with the arbuscular mycorrhizal fungus *Rhizoglomus irregulare* under low phosphorus (P) availability (0.47 mg kg^-1^). Transcripts were normalized against the *ZmActin1* gene. For each *Pht1* gene, Welch’s statistics and P values are reported. In columns, values followed by the same letter do not differ significantly at P ≤ 0.05 by Tamhane T2 test.

| **Maize line** | **ZmPHT1;1** | **ZmPHT1;3** | **ZmPHT1;4** | **ZmPHT1;5** | **ZmPHT1;8** | **ZmPHT1;9** | **ZmPHT1;11** | **ZmPHT1;12** |
| --- | --- | --- | --- | --- | --- | --- | --- | --- |
| **B73** | 0.59±0.25 a | 0.12±0.05 a | 1.07±0.23 d | 0.68±0.52 b | 0.35±0.01 c | 0.80±0.35 a | 0.16±0.12 b | 0.06±0.03 b |
| **Mo17** | 0.93±0.26 a | 0.37±0.21 a | 67.11±4.82 a | 10.32±0.19 a | 14.99±0.63 a | 1.01±0.30 a | 5.96±0.72 a | 2.47±0.63 a |
| **Oh40B** | 0.53±0.13 a | 0.07±0.01 a | 3.39±0.33 c | 1.56±0.17 b | 2.84±0.30 b | 0.54±0.02 a | 0.60±0.17 b | 0.29±0.16 b |
| **Oh43** | 1.00±0.25 a | 0.64±0.31 a | 14.88±3.80 b | 2.06±0.87 b | 1.92±0.37 b | 1.16±0.28 a | 0.69±0.29 b | 0.38±0.22 b |
| **Welch’s** | 2.33 | 4.41 | 79.15 | 258.69 | 114.74 | 4.57 | 44.62 | 12.51 |
| **d.f.** | 3,10.5 | 3,8.3 | 3,9.9 | 3,9.9 | 3,8.7 | 3,8.5 | 3,10.2 | 3,8.6 |
| **P** | 0.13 | **0.04** | **<0.001** | **<0.001** | **<0.001** | **0.03** | **<0.001** | **0.002** |

**Table S3.** Normalized expression values (mean ± standard error of the mean, the displayed values are multiplied by 10^3^) of *Rhizoglomus irregulare* phosphate transporters (*RiPT*s) in extraradical (ERM) and intraradical (IRM) mycelium developed in symbiosis with *Zea mays* plants under low phosphorus (P) availability (0.47 mg kg^-1^). Transcripts were normalized against the *ef1-α* gene. Welch’s statistics and P values are reported for each *RiPT* gene and maize line. In columns and for each fungal mycelium type, values followed by the same letter do not differ significantly at P ≤ 0.05 by Tamhane T2 test.

| **Maize line** | **ERM *RiPT1*** | **ERM *RiPT2*** | **ERM *RiPT3*** | **ERM *RiPT5*** | **ERM *RiPT7*** |
| --- | --- | --- | --- | --- | --- |
| **B73** | 0.06 ± 0.02 | 2.55 ± 0.52 | 3.52 ± 0.94 | 0.66 ± 0.15 | 22.86 ± 1.11 |
| **Mo17** | 0.29 ± 0.17 | 20.05 ± 3.57 | 9.72 ± 4.22 | 1.08 ± 0.18 | 68.23 ± 11.05 |
| **Oh40b** | 0.29 ± 0.05 | 9.96 ± 0.82 | 6.61 ± 0.96 | 1.56 ± 0.31 | 51.64 ± 1.77 |
| **Oh43** | 0.21 ± 0.03 | 11.66 ± 1.20 | 12.06 ± 2.92 | 0.48 ± 0.07 | 30.83 ± 3.68 |
| **Welch’s** | 10.76 | 30.80 | 3.50 | 5.69 | 58.95 |
| **d.f.** | 3,9.8 | 3,10.1 | 3,10.3 | 3,9.9 | 3,9.9 |
| **P** | **0.002** | **<0.001** | 0.056 | 0.16 | **<0.001** |
| **Maize line** | **IRM *RiPT1*** | **IRM *RiPT2*** | **IRM *RiPT3*** | **IRM *RiPT5*** | **IRM *RiPT7*** |
| **B73** | 0.50 ± 0.12 | 23.15 ± 7.49 | 7.52 ± 0.76 | 0.47 ± 0.13 | 50.90 ± 5.89 |
| **Mo17** | 0.57 ± 0.08 | 26.56 ± 3.73 | 14.57 ± 5.18 | 0.87 ± 0.26 | 76.74 ± 15.21 |
| **Oh40B** | 0.71 ± 0.11 | 61.92 ± 18.64 | 12.46 ± 2.25 | 0.82 ± 0.36 | 69.77 ± 17.27 |
| **Oh43** | 0.62 ± 0.10 | 26.56 ± 4.37 | 7.89 ± 0.88 | 0.95 ± 0.19 | 69.55 ± 20.26 |
| **Welch’s** | 0.54 | 1.12 | 1.75 | 1.54 | 1.08 |
| **d.f.** | 3,10.9 | 3,10.4 | 3,10.2 | 3,10.5 | 3,9.7 |
| **P** | 0.66 | 0.38 | 0.22 | 0.26 | 0.40 |

**Table S4.** Results of one-way ANOVA comparing normalized values of *Rhizoglomus irregulare* phosphate transporters (*RiPT*s) expression in extraradical (ERM) and intraradical (IRM) mycelium developed in symbiosis with *Zea mays* plants under low phosphorus (P) availability (0.47 mg kg^-1^). F and P values are reported for each PT gene and maize line.

|  | **One way ANOVA: IRM vs ERM *RiPT* expression levels** | | | | |
| --- | --- | --- | --- | --- | --- |
| **Maize line** | ***RiPT1*** | ***RiPT2*** | ***RiPT3*** | ***RiPT5*** | ***RiPT7*** |
| **B73**  **F_1,4_**  **P** | 14.29  **0.02** | 18.42  **0.01** | 6.61  0.06 | 0.53  0.50 | 48.02  **0.002** |
| **Mo17**  **F_1,4_**  **P** | 2.10  0.22 | 2.23  0.22 | 0.51  0.51 | 0.43  0.55 | 0.29  0.62 |
| **Oh40b**  **F_1,4_**  **P** | 9.77  **0.03** | 9.27  **0.04** | 4.82  0.09 | 2.70  0.22 | 0.47  0.52 |
| **Oh43**  **F_1,4_**  **P** | 46.52  **0.002** | 12.91  **0.02** | 3.36  0.14 | 4.75  0.09 | 1.68  0.26 |

**Table S5.** Percentages of arbuscules, vesicles and hyphae occurring in the roots of the four maize inbred lines. F and P values from one way ANOVA are reported for each fungal structure detected in colonized roots.

| **Maize line** | **Arbuscules**  **(%)** | **Vesicles**  **(%)** | **Hyphae**  **(%)** |
| --- | --- | --- | --- |
| **B73** | 32.6 ± 5.9 | 29.0 ± 3.3 | 19.3 ± 3.7 |
| **Mo17** | 29.1 ± 4.2 | 15.1 ± 4.9 | 16.8 ± 3.8 |
| **Oh40b** | 18.5 ± 5.2 | 19.7 ± 3.3 | 25.2 ± 3.9 |
| **Oh43** | 25.7 ± 2.0 | 26.2 ± 4.4 | 13.5 ± 1.7 |
| **ANOVA** |  |  |  |
| **F_3,12_** | 1.73 | 2.44 | 2.15 |
| **P** | 0.21 | 0.11 | 0.15 |
